# Assessing a Digital Tool to Screen and Educate Survivors of Domestic Violence on Affordable Housing Programs in New York City: Protocol for a Mixed Methods Feasibility Study

**DOI:** 10.2196/63162

**Published:** 2025-05-02

**Authors:** Jennifer K Tan, Michelle R Kaufman

**Affiliations:** 1 Department of Health, Behavior and Society Johns Hopkins Bloomberg School of Public Health Baltimore, MD United States; 2 Department of International Health Johns Hopkins Bloomberg School of Public Health Baltimore, MD United States

**Keywords:** domestic violence, intimate partner violence, homelessness, housing, feasibility study, digital intervention, digital solution

## Abstract

**Background:**

Extant research has long documented the association between domestic violence and homelessness. Yet, there appear to be few interventions to address the housing needs of survivors of domestic violence, and none on a digital platform.

**Objective:**

Our primary objective is to determine the feasibility of a full-scale intervention trial of a web-based tool that screens and educates survivors of domestic violence on affordable housing programs in New York City. Our secondary objectives are to assess the perceived usability and acceptability of the tool.

**Methods:**

The study will take place in a community-based domestic violence center in New York City. Treatment will consist of study participants not using (SC) or using (SC+) the tool, in or outside of private meetings with a case manager to discuss housing and other benefits. The frequency of the meetings will vary depending on the participant’s needs. The study will measure changes in housing knowledge, housing self-efficacy, and staff trust through two electronic surveys, administered at times 0 and 2 weeks. Following a historical cohort control group design, we will sequentially recruit participants, starting with SC and followed by SC+. After data collection for SC+ ends, we will invite staff from the partner site to individual, web-based interviews to share their experiences of and recommendations for implementing the tool.

**Results:**

Recruitment for the SC arm commenced in March 2022 and was completed in April 2023. After a year, 23 participants completed the study: 75 were screened, 44 were deemed eligible, 35 enrolled, and in the end, 23 participants completed baseline and follow-up surveys. Given the length of time it took to recruit for SC and the limited time overall that we had for the study, the study team decided to follow an expedited recruitment timeline for SC+. Recruitment for SC+ commenced in January 2024 and is anticipated to end by May 2024. Recruitment for the staff interviews will take place in June 2024. We expect to complete the study and be ready to compile the results by the end of June 2024.

**Conclusions:**

The protocol describes a feasibility study that can inform future research on housing or digital tools for a similar study population. Data from the study will also be used to inform revisions to the tool.

**International Registered Report Identifier (IRRID):**

DERR1-10.2196/63162

## Introduction

### Background

Survivors of domestic violence (survivors) in the United States are at heightened risk of homelessness [1,2]. Homelessness refers to a state in which an individual does not have a fixed place to stay or is living in a place unsuitable for human habitation [3]. In New York City, more than 2 in 5, or 41%, of families entering the City’s homeless shelter system, run by the Department of Homeless Services (DHS), have attributed their homelessness to domestic violence [4]. Short-term emergency shelters, specifically for domestic violence in New York City, offer survivors much critical aid; however, one study found that one-quarter of survivors exiting from emergency shelters moved directly into DHS shelters [5]. Presently, over 86,000 individuals are served by DHS shelters on a given day [6], up from prepandemic estimates that hovered closer to 60,000 [4]. Against a backdrop of about 600 domestic violence–related calls to the New York Police Department a day [7], the numbers, alone, underscore the significance of homelessness and domestic violence in New York City. The research team of this feasibility study thus purposively selected New York City as the study setting given its disproportionate need.

Remarkably, considering the sheer magnitude of domestic violence and its bidirectional relationship with homelessness in the United States and New York City, few housing solutions have been designed to specifically address the housing needs of survivors of domestic violence. The few solutions that follow, however, show promise.

### Temporary Housing

Two common forms of temporary housing are emergency shelters and transitional housing. Emergency shelters are a type of homeless shelter allocated for use by survivors of domestic violence. In New York City, emergency shelters provide survivors with a temporary place of stay, for 90 days initially, with a possible extension of up to 180 days [8]. Transitional housing in New York City, likewise, offers survivors a temporary place of stay, for up to 2 years in facility-based or scattered-site programs. In facility-based programs, survivors reside in the same building; whereas in scattered-site programs, survivors receive rental subsidies to live in apartment sites scattered throughout the community or in their current homes if the abuser has vacated the home [9].

While little research to the best of our knowledge exists on temporary housing’s housing outcomes, prior research in the United States does suggest that emergency shelters and transitional housing lead to positive safety outcomes for survivors [10,11]. According to Messing et al [10], female survivors who left their perpetrators for emergency shelter experienced significantly less abuse during the follow-up period of 8 months than female survivors who did not seek emergency shelter. Findings from another study indicated that a longer shelter stay led to a lower degree of repeat abuse in the initial 6 months following a survivor’s exit from the shelter [11]. Although these studies did not take place in New York City, we can infer from their findings that emergency shelters and transitional housing can contribute to promising safety outcomes for survivors in New York City.

### Permanent Supportive Housing

Permanent supportive housing (PSH) programs offer individuals and families a government-subsidized place of stay, for which tenants typically contribute one-third of their income to rent [12], along with a broad range of support services to help tenants with their clinical and nonclinical needs [13]. In the United States, PSH’s benefits have included reductions in emergency and hospital use and substance use, and fewer encounters with the criminal justice system [14]. While PSH programs are available to and accessed by survivors in New York City, many have not been designed specifically for the needs of survivors. Many PSH programs, for instance, do not include safety planning, a critical strategy for survivors leaving their abusers [9].

Housing First (HF) [15] is a type of PSH program that prioritizes rapid housing placement. The HF model hypothesizes that helping someone obtain stable housing first will facilitate their subsequent ability to address other concerns; hence, HF programs seek to connect tenants with permanent, independent housing first, prior to addressing other issues [15,16]. Based on HF’s successes with adults with mental illness and substance abuse issues, agencies across the United States are now exploring adaptations of HF for domestic violence. One such successful adaptation has been the Washington State Domestic Violence Housing First Program (DVHF) [16]. Designed with survivors’ needs in mind, the DVHF includes safety planning, as well as various support services, including home security and lock changes, counseling, career training, and legal services. Initial results have been promising. In the inaugural cohort of four agencies, the DVHF saw 89% of survivors retain, that is, maintain or obtain permanent housing by the end of the 18-month study period, as compared with one-third at the start of the study [17]; similarly, in the second cohort of nine agencies, the DVHF saw 88% of survivors retain housing by the end of the 36-month study period, as compared with about half at the start of the study [18]. In addition, DVHF in both studies led to increases in survivor-reported independence and positive shifts in needs and priorities [17,18].

Presently, there are no HF programs in New York City tailored for survivors of domestic violence. However, the promising performance of DVHF programs in states like Washington offers encouragement for their adoption in places like New York City.

### Rapid Rehousing and Flexible Funding

Besides DVHF, rapid rehousing and flexible funding are two other promising innovative models. Rapid rehousing refers to an approach consisting of an intensive housing placement effort including housing identification, a time-limited subsidy, and additional education and support to retain housing. Flexible funding refers to an approach in which domestic violence agencies may grant survivors discretionary funding for basic needs, like changing locks, moving, storage, or security deposits.

While research on these newer approaches has been sparse, a few published studies have supported their potential in helping survivors access and retain housing in the United States. In one study, 94% of survivors in an urban city who were about to lose housing at the start of the study reported positive housing outcomes just 6 months after receiving flexible funding [19]. A rapid rehousing pilot, moreover, found that, in comparison with participants given standard shelter services, rapid rehousing participants saw a reduction in the length of shelter stay and recidivism back to shelter [20]. While we are not aware of any peer-reviewed program evaluations in New York City, rapid rehousing and flexible funding both saw upticks in use among New York City–based domestic violence agencies during the COVID-19 pandemic [21,22].

### Gaps to Be Addressed

Currently, when a survivor asks their case manager, “What are my housing options?” and “What am I eligible for?” the case manager would typically collect information about the survivor, manually determine which options would work best and are available, explain the options to the survivor, and then apply for each option with the survivor. The process is arduous and could take a few months. In parallel, the survivor may receive different information from different sources, further complicating the process and leading the survivor to feel overwhelmed.

While the existing housing solutions, albeit sparse, have shown promise, there appears to be no digital solution that survivors can use to address their housing needs. This is especially salient given most adults (90%) in the United States, regardless of socioeconomic status, have smartphones, and more than half with browsing capability [23,24]. Digital solutions furthermore have served as promising complements to traditional services like counseling and advocacy [25-27]. Thus, there is an opportunity to develop a digital tool to address the housing needs of survivors.

### Description of the Tool

**Figure 1:**
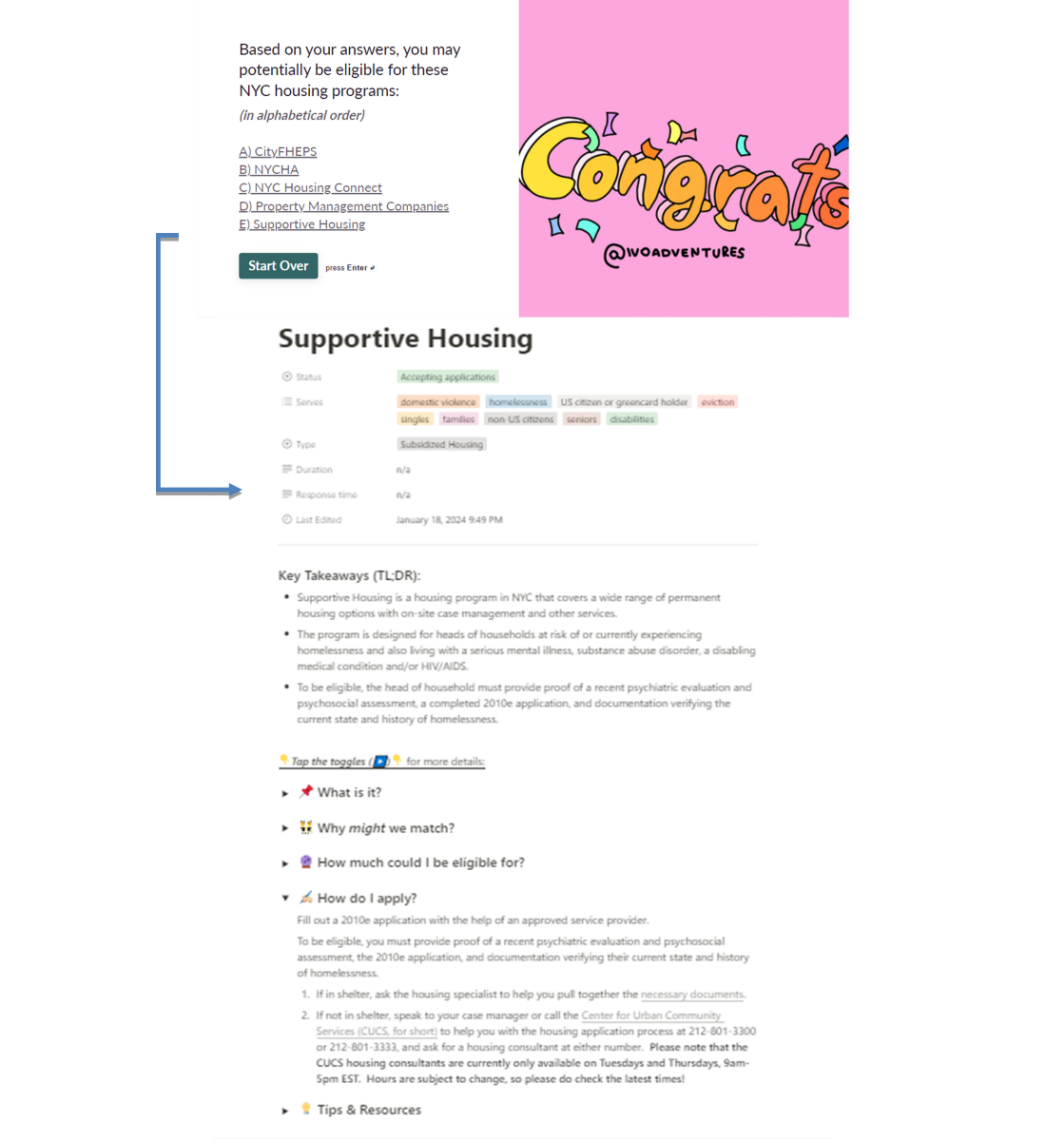
Snapshots of tool.

Knowing the above, the study team collaborated with a coalition of domestic violence housing specialists in New York City to create a web-based tool that both screens and educates survivors on affordable housing programs in New York City. Grounded in the process of empowerment [28], the digital tool strives to address survivors’ barriers to housing knowledge while building survivors’ self-efficacy to achieve their housing goals. Taking only a few minutes, the tool starts by collecting survivors’ household size, income, and living situation, and ends by generating curated matches of housing programs based on the survivors’ information (Figure 1). The tool gives the survivor the agency to determine and learn about their options, without the case manager serving as the sole gatekeeper of information. Because it is one tool, the survivor and case manager also reference the same information, thereby, standardizing the process and freeing up the case manager and survivor to focus on applying for housing programs.

It is important to note that the tool neither guarantees the survivor’s access to housing programs nor addresses every housing need of every survivor. Still, the tool introduces survivors to relevant, available housing programs, all from the convenience of their phones. The tool also serves survivors and nonsurvivors alike. Its content on housing programs is broadly relevant to anyone keen to determine their eligibility for affordable housing programs in New York City.

For this feasibility study, the study team will test the concept of the tool with a convenience sample of survivors served by a community-based domestic violence center (site) in New York City. The site serves a wide range of clients and is particularly attentive to the needs of survivors with disabilities.

### This Study

This paper presents the protocol for testing the concept of the tool in a community-based domestic violence center. The feasibility study aims to determine whether implementing a web-based tool in this setting is possible; and whether the delivery of housing information through a digital tool could show promise of increasing survivors’ housing knowledge, housing self-efficacy, or trust in staff. In this paper, we use the terms “full-scale” or “large-scale” trials to denote next-phase trials that will be fully powered to measure impact. This study will compare the intervention with a control condition of no intervention use. We hypothesize that the intervention group will report greater increases in at least one of the primary outcomes: housing knowledge, housing self-efficacy, and staff trust, relative to the control condition. We also hypothesize that most survivors using the intervention will rate the tool as usable and acceptable.

### Research Questions

Through this study protocol, we are seeking to answer the following research questions: (1) What is the initial treatment effect of SC+ (standard care plus), as compared to SC (standard care), on survivors’ housing knowledge, housing self-efficacy, and staff trust? (2) What is the estimated sample size needed for a full-scale intervention trial? What is the estimated timeline needed to reach this sample size? (3) Are the expected rates of recruitment and retention achieved? (4) Are the study’s processes and instruments feasible for a full-scale intervention trial? and (5) Is the tool acceptable and usable by survivors and staff?

## Methods

### Study Design

The study will use a quasi-experimental, mixed methods design to assess the feasibility, usability, and acceptability of the tool (Multimedia Appendix 1). There will be two treatment groups: no use of the tool (standard care only, SC) and use of the tool (standard care plus, SC+). Survivors mostly come to the site through the organization’s crisis hotline. A survivor calls the hotline for help, and if deemed appropriate, the site makes an appointment for an intake interview to determine the survivor’s needs, including housing. Subsequently, a case manager is assigned to meet with the survivor. For new clients, the meetings normally take place twice a week during their first month. In these meetings, the case manager helps the survivor apply for a myriad of benefits, including housing programs if housing has been identified as a need. The meeting frequency then lessens as the survivor’s needs are met. SC will consist of no tool use, in or outside of the case manager meetings. SC+ will consist of tool use, in or outside of the case manager meetings. To ensure access to the tool regardless of whether a meeting is scheduled during the study period, the study team will email all SC+ participants a link to the tool for their self-use.

Following a historical cohort control group design, the study team will recruit participants into each arm, sequentially. We will recruit SC participants first. After all SC participants have completed their follow-up surveys, SC+ recruitment will begin. The SC and SC+ study groups will engage in two data collection points: one baseline survey prior to the housing meeting and one follow-up survey 2 weeks after completion of the baseline survey, each anticipated to take up to 30 minutes to complete. Study participants will be given a 1-week grace period to complete each survey. During SC+, the study team will email the tool to all enrolled participants following the completion of their first survey.

After SC+, we will engage five staff participants (key informants) in 60-minute individual interviews through a web conferencing platform like Zoom (Zoom Communications, Inc) about their experiences using the tool. The staff serve as decision-makers who can influence whether the tool will be used after the study. Thus, their perspectives are critical for understanding how to successfully implement the tool in the partner site and similar settings. Only 5 of the 12 staff met the eligibility criteria, which were determined in consultation with the partner site. We will not engage survivors in interviews to mitigate potential research fatigue [29]. We will, however, ask survivor participants about their experiences using the tool in the SC+ follow-up survey.

The study team had initially considered a randomized controlled trial (RCT) but selected a quasi-experimental, historical cohort design for several reasons. First, randomizing at the participant level, as in an RCT, was not feasible. Randomizing participants would have required the case managers to know the participant’s enrollment status and arm assignment to introduce the tool to the appropriate person. This lack of concealment could have produced differential treatment by the case managers based on the participant’s enrollment status. Second, we anticipated that tracking each participant’s arm assignment in an RCT would have added to the staff’s already limited capacities, a concern that the site also shared. Third and final, there was the concern that SC+ participants could interact with SC participants and share the tool, further confounding the results. Fortunately, the current historical cohort control group design mitigates the above risks. In the current design, case managers deliver SC, followed by SC+, regardless of whether a participant enrolls, thereby reducing any potential administrative burden on the staff. While the case managers are not entirely blinded to the participants’ enrollment status and arm assignment, the current design reduces the risk by emailing participants directly with the intervention link. If a participant mentions the link to the case manager, then the case manager will know that they have signed up for SC+. Otherwise, case managers will not know and will be encouraged to share the tool with all clients. Furthermore, the tool will only be accessible to participants during the SC+ arm, hence removing the possibility of participants interacting with the tool during the SC arm. To mitigate ethical concerns over one group and not the other receiving the tool, all SC participants will receive access to the tool after the SC+ arm completes data collection. Thus, while the study team had considered an RCT, the quasi-experimental, historical cohort control group design ultimately allowed the study team and partner site greater logistical flexibility, decreased administrative burden, and lower risk of confounding.

### Study Population

The partner site, a community-based domestic violence program based in New York City, provides domestic violence and sexual assault counseling, case management, legal services, occupational therapy, and support groups for any survivor of domestic violence and is particularly attuned to the needs of survivors who are D/deaf or Hard of Hearing or have a disability [30]. Based on the last set of data collected by the site in 2021, the partner site, on average, served 171 new and existing clients per month. About 26% (n=45) of clients were new to the program each month. Of those who disclosed their information to the site, 84% (109/130) identified as a cisgender woman, and 15% (19/130) as a cisgender man. The majority identified as either Black (48/114, 42%) or Latino/a/x (32/114, 28%). None chose to disclose their employment status, but more than half (76/151) reported being on some form of public assistance. Finally, 52% (66/128) of clients reported a mental health disability, 18% (23/128) reported a sensory disability (ie, blindness or deafness), 17% (22/128) a physical disability, 10% (13/128) a medical condition, and 3% (4/128) a cognitive disability. The site has three full-time case managers, who cover a wide range of survivor needs including housing, and a full-time program director and assistant director who oversee the case managers.

### Eligibility Criteria

Survivors, or participants, will be eligible for study inclusion if they consent to participate; are seeking assistance from the site at the time of joining the study; are at least 18 years old; have access to a device with web-browsing capability (eg, smartphone, tablet, or the site’s computers); are looking for housing; have a safe email account, with “safe” being interpreted as the survivor sees fit; can read English without assistance from others including translation or interpretation assistance; and have not participated in the study before. Staff, or key informants, will be eligible for inclusion in the study if they give oral consent to participate, are at least 18 years old, and bear the title or responsibilities of “case manager” or “director.” We expect to recruit five key informants.

### Procedures

**Figure 2:**
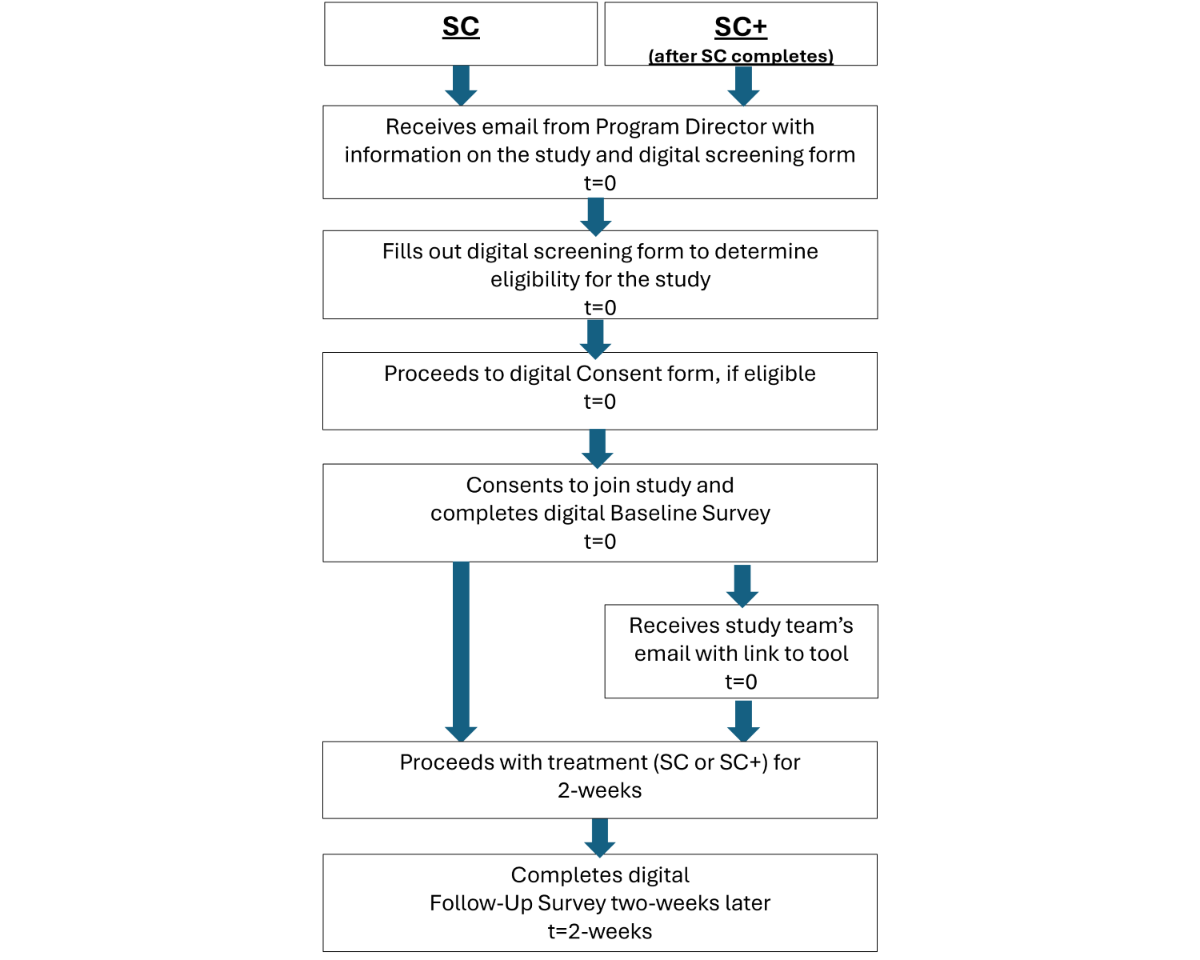
Participant flow. SC: standard care only; SC+: standard care plus.

The two sequential study arms will follow the same procedures, differing only in treatment (Figure 2). In each arm, the program director will send a mass email about the study to their clients with the recruitment flyer and screening form link. The case managers may also refer prospective participants to the study in their individual meetings. If the prospective participant is interested, they will answer questions on a digital form to determine whether they meet the inclusion criteria. If the prospective participant meets all the inclusion criteria, the form will automatically collect their contact information, which will then trigger a digital consent form that will be emailed to the participant. If the prospective participant wishes to join the study after reading the consent form, they will click yes to proceed to the baseline survey. As we are waiving the collection of a signature to protect the participant’s confidentiality, clicking yes will constitute the participant’s consent to join the study. Should the participant proceed, they will receive the baseline survey immediately and a follow-up survey 2 weeks later, both by email. Each survey will be active for 1 week, and up to 3 email reminders will be sent to the participant during the 1-week grace period if the survey remains incomplete. If the participant completes the follow-up survey during the grace period, they will receive an additional email upon completion with the answer key to the knowledge quiz.

During the 2-week period between the baseline and follow-up surveys, the participant will receive services as usual in the SC arm and services plus the tool in the SC+ arm. Regardless of whether a participant completes the surveys, the site will continue to provide services to the participant. In the SC+ arm, the study team will email the participant a link to the tool to use over the 2-week treatment period. The case manager will also share the tool in their individual meetings with SC+ participants. If there happens to be no meeting during a participant’s enrollment in the study, the participant can still access the tool through the study team’s email.

For each completed survey, the participant will receive a US $10 Visa eGift card via email for a potential total of US $20 for two completed surveys. Study data will be collected and managed using REDCap (Research Electronic Data Capture; Vanderbilt University) electronic data capture tools hosted at Johns Hopkins University [31,32]. REDCap is a secure, web-based software platform designed to support data capture for research studies, providing (1) an intuitive interface for validated data capture, (2) audit trails for tracking data manipulation and export procedures, (3) automated export procedures for seamless data downloads to common statistical packages, and (4) procedures for data integration and interoperability with external sources.

### Outcome Measures

#### Overview

For all measures (Table 1), the time frame will be the current point-in-time at which the participant is answering the survey. It is important to note that the measures do not presently pertain to specific types of disabilities, thereby potentially limiting our ability to relate the findings to participants with disabilities. Still, the measures and subsequent findings can be used to inform future studies engaging survivors of domestic violence, generally, and serve as a base for the inclusion of questions specific to survivors with disabilities.

**Table 1:** Research questions, outcome measures, and time of data collection.

	Research question	Audience		Time and method of data collection		
		Survivor	Staff	Baseline survey	Follow-up survey	Individual interview
1	What is the initial treatment effect of SC+^a^, as compared to SC^b^, on survivors’ housing knowledge, housing self-efficacy, and staff trust?	✓		✓	✓	
2	What is the estimated sample size needed for a full intervention trial? What is the estimated timeline needed to achieve the full intervention trial sample size?	✓				
3	Are the expected rates of recruitment and retention achieved?	✓				
4	Are the study’s processes and instruments feasible for a full intervention trial?	✓		✓	✓	✓
5	Is the tool acceptable and usable by survivors and staff?	✓	✓		✓	✓

^a^SC+: standard care plus.

^b^SC: standard care only.

#### Sociodemographics

The study will collect sociodemographic information from participants at baseline. Variables include age, gender, income, length of time with the site, prior or current domestic violence exposure, housing status, and experiences of housing instability in the last 6 months.

#### Housing Knowledge

As no known prior studies have measured housing knowledge, we will assess whether the tool improves survivors’ knowledge of housing options through a 10-item Housing Knowledge Quiz developed for this study (Table 2). Questions like “Which housing program is a lottery of affordable housing apartments in New York City?” will ask survivors about affordable housing programs in New York City and program eligibility criteria, with each question carrying a 1-point weight for a possible summed score range of 0 to 10.

**Table 2:** Housing knowledge quiz.

Question	Answer choices
1. Jane is a survivor of domestic violence in shelter. She and her two children plan to move out of shelter to an apartment. What is Jane’s household size?	1,2,3
2. “Affordable housing” means that you pay about one-third or less of your income on rent.	True, False
3. Eligibility for New York City housing programs can depend on your...	Household size, Income amount, Citizenship or residency status, Current living situation, Whether you have children or are expecting children, Disability, All of the above
4. Which housing program is a lottery of affordable housing apartments in New York City?	FHEPS^a^, Housing Connect, Property Management Companies, Do Not Know. Please select one answer.
5. Which housing program lists apartment openings, reviews housing applications, and takes payment?	FHEPS, Housing Connect, Property Management Companies, Do Not Know. Please select one answer.
6. Which housing program is a type of rental assistance paid directly by the government to the landlord? The amount of rental assistance will vary by one’s income and household size.	FHEPS, Housing Connect, Property Management Companies, Do Not Know. Please select one answer.
7. DHS^b^ shelter provides a time-limited stay only for households of domestic violence.	True, False
8. Supportive housing is a type of permanent housing that also includes services like case management, medical, and psychiatric help.	True, False
9. Who may be eligible for SSI^c^ benefits?	Individuals or couples who are 65 years or older, blind, or disabled, with limited income and resources, Children who are disabled or blind, with limited income and resources, All of the above, None of the above
10. What is the name of a property management company that manages affordable and low-income housing in New York City?	Reliant Realty, Insignia, Barrier Free Living, Urban Resource Institute. Please select one answer.

^a^FHEPS: family homelessness and eviction prevention supplement.

^b^DHS: Department of Homeless Services.

^c^SSI: supplemental security income.

#### Housing Self-Efficacy

According to social cognitive theory, the stronger one’s self-efficacy, the higher the goals one sets and the more favorable the outcomes one expects, and vice versa [33]. In Cattaneo and Chapman’s [28] empowerment process model, likewise, self-efficacy drives the rest of the components: power-oriented goals, knowledge, competence, action, and impact. To measure whether the tool improves survivors’ abilities to achieve their housing goals, we have constructed a 10-item Housing Self-Efficacy Likert scale (Table 3), adapted from three existing scales to incorporate housing: the Generalized Self-Efficacy Scale [34], the Financial Self-Efficacy Scale [35], and the Scale of Economic Self-Efficacy [36]. The questions have been constructed using 4 response categories, from 1=strongly disagree to 4=strongly agree. Participants will be asked to rate their level of agreement on questions such as “I can always manage to solve difficult housing problems if I try hard enough” and “If I experience housing trouble, I can usually think of a solution.” To balance the potential “forced-choice” nature of the four categories, a “does not apply” option has also been added.

**Table 3:** Housing self-efficacy scale.

No.	Item
1	I can always manage to solve difficult housing problems if I try hard enough.
2	If I have a housing problem, I can find ways to get what I need.
3	It is easy for me to stick to and accomplish my housing goals.
4	I am confident that I could deal efficiently with unexpected housing events.
5	Thanks to my resourcefulness, I know how to handle unforeseen housing situations.
6	I can solve most housing problems if I invest the necessary effort.
7	I can remain calm when facing housing difficulties because I can rely on my abilities.
8	When I am confronted with a housing problem, I can usually find several solutions.
9	If I experience housing trouble, I can usually think of a solution.
10	No matter what housing problem comes my way, I am usually able to handle it.

#### Staff Trust

Trust is defined as an individual’s expectation that their needs will be met without any harm ​[37]​. When developing the tool, the coalition of housing specialists continually expressed an interest in knowing how the tool would affect survivor trust in staff. For them, they hoped the tool could validate and explain the staff’s housing advice to survivors. An abundance of research further illustrates the importance of trust in service delivery. Patient trust in health care settings, for instance, has been correlated with patient satisfaction and continuity of care [38-41]​. While several valid, reliable scales have been designed to measure trust in service delivery ​[38,42,43], no trust scale quite captures the uncertainty of the emergency shelter context or the often-volatile relationship between staff and survivors. The 10-item Trust in Community Health Workers (CHWs) Scale ​[37]​ was thus adapted to measure survivors’ perceptions of staff trustworthiness, particularly of their case managers. CHWs in low- and middle-income countries are comparable to case managers in domestic violence agencies in that both typically work with vulnerable groups in resource-constrained, emergency settings. We adapted the Trust in CHWs Scale then, not only for its validity and reliability but also for its applicability to an emergency setting, like that of a domestic violence center. Like the Housing Self-Efficacy Scale, the resulting Staff Trust Scale (Table 4) will use 4 response categories, from 1=strongly disagree to 4=strongly agree, along with a “does not apply” category for additional scenarios. Sample questions include, “My case manager tells me everything I need to know when dealing with my housing problems” and “In my opinion, my case manager is committed to providing me with the best care possible.”

**Table 4:** Staff Trust Scale.

No.	Item
1	My case manager tells me everything I need to know when dealing with my housing problems.
2	My case manager knows as much as they can about finding housing.
3	My case manager takes enough time with me during our housing meetings.
4	My case manager keeps what we discuss confidential or private from others at [agency name].
5	In my opinion, my case manager is committed to providing me with the best care possible.
6	My case manager is an excellent listener.
7	I feel better emotionally after talking to my case manager.
8	My case manager treats me with respect.
9	My case manager has my best interest at heart.
10	My case manager makes me feel like I am worthy of their time.

#### Sample Size, Recruitment and Retention Rates, and Timeline

We will estimate the desired sample size for a full-scale trial based on the effect sizes achieved in this study in the housing knowledge, housing self-efficacy, and staff trust variables. Both the rates of recruitment and retention will be measured quantitatively; the recruitment rate will be defined as the number of participants who enroll divided by the total number of participants recruited, while the retention rate will be defined as the number of participants who complete the follow-up survey divided by the number of participants who complete the baseline survey. Finally, the timeline, or the time it takes to recruit participants, will be recorded to give a benchmark for the full-scale intervention trial.

#### Large-Scale Trial Feasibility

To determine the feasibility of a full-scale trial, we will look at response rates, ease of implementation, and outcome appropriateness, as well as learn how participants respond to the instruments. The response rate on the surveys will serve as a proxy of instrument feasibility and will be calculated as the number of items answered on a survey divided by the total number of survey questions. A “does not apply” answer will count as an item nonresponse, or unanswered. If a 75% response rate or higher is achieved, then the instrument will be deemed feasible. A list of the questions that were answered versus unanswered will also be compiled to improve the instruments.

Ease of implementation, on the other hand, will be measured solely in the key informant interviews. Staff will be asked open-ended questions on their experiences with participant recruitment and use of the tool, for example, “What were some facilitators or challenges to recruiting participants?” “...to the use of the tool in your meetings?”

Perceived outcome appropriateness, finally, will be measured qualitatively through questions on the SC+ follow-up survey and informant interviews on whether housing knowledge, self-efficacy, and staff trust were appropriate to measure for the tool, as well as whether participants recommended other outcomes to measure.

#### Acceptability and Usability of the Tool

To understand the survivors’ and staff’s experiences of using the tool, we will collect data from the survivors through surveys (Table 5) and the staff through interviews (Textbox 1).

**Table 5:** Perceived Usability Scale.

	Items
1	The tool is easy to use.
2	I had no trouble seeing the information in the tool.
3	The information in the tool is easy to understand.
4	The information in the tool is relevant to me.
5	I found the tool hard to use.

The survivors’ and staff’s intent to continue use and perceived effect will serve as proxies of the tool’s acceptability. In the follow-up survey to the SC+ group of survivors, intent to continue use will be measured by participants’ responses to the statements, “I plan to continue using the tool,” and “I would recommend the tool to others in a similar situation as me,” based on their tool use from the prior 2 weeks. For each statement, participants will also have an opportunity to elaborate on their answer with the open-ended question “Why?” Similarly, in the key informant interviews, staff will be presented with open-ended questions: “How likely are you to continue using the tool or recommend the tool to other case managers beyond the feasibility study? Why or why not?” To gauge the perceived effect, staff will be encouraged to identify changes in their workloads, relationships with survivors, and the survivors’ housing search approach after using the tool with questions like, “How does using the tool impact your or your staff’s workload? Do you have examples?” and “Have you noticed changes in your relationship with survivors as a result of using the tool?”

Perceived usability, on the other hand, will be measured through 5 items in the SC+ follow-up survey to survivors, as well as open-ended interview questions to the staff on navigation and content relevance. The 5-item measure to survivors was adapted from a 16-item Likert scale used to evaluate the bMORESafeapp [25] and has a response set ranging from 1=strongly disagree to 4=strongly agree, with a “does not apply” category for additional scenarios. Only five items of the original 16-item Likert scale measured general app usability. The remaining 11 items were specific to features found only in the bMORESafeapp.

Finally, the staff will be asked for their input on the future dissemination of the tool within the partner site and to other agencies through open-ended questions, like “How could the tool be used by others (eg, housing specialists, case managers, social workers, lawyers, real estate brokers, and other domestic violence advocates)?”

**Key informant interview guide (outcome and questions).**

**Introduction**

- Tell me about yourself.
- How long have you worked at the partner site?
- What is your role at the site? What does that entail?
- What are you most proud of in your work?

**Intent to continue use (acceptability)**

- How likely are you to continue using the tool with clients beyond the feasibility study? Why or why not? Scale of 1-4, 1=strongly disagree, 4=strongly agree.
- How likely are you to recommend the tool to other case managers beyond the feasibility study? Why or why not? Scale of 1-4, 1=strongly disagree, 4=strongly agree.

**Perceived effect (acceptability)**

- How has using the tool impacted your or the staff’s workload? Do you have examples?
- Was there something that you learned from the tool that you think is valuable to your work? To your relationship with clients?
- Have you noticed any changes in your or the staff’s relationship with clients as a result of using the tool?
- Have you noticed any changes in how clients have approached their housing search as a result of using the tool?

**Ease of implementation (implementation)**

- What were some facilitators or challenges to recruiting participants?
- What were some facilitators or challenges to the use of the tool in your meetings? (eg, leadership support, internet stability, tech comfort, meeting duration, and client disinterest)

**Outcome appropriateness (implementation)**

- Were the outcomes of knowledge, self-efficacy, and staff trust appropriate for the tool?
- Would you add other outcomes to consider? If so, what would they be, and why?

**Perceived usability**

- Can you describe how (easy or difficult) it was to navigate the tool? See the information in the tool? Guide clients on how to use the tool?
- How relevant was the information presented by the tool to your discussions with clients?
- Did any of the content help start a conversation? Why or why not?
- How would you explain the tool to a client? To another staff member?

**Dissemination**

- What suggestions do you have for disseminating the tool to staff and clients in the rest of the organization? Other agencies?
- How could the tool be used by others (eg, housing specialists, case managers, social workers, lawyers, real estate brokers, and other domestic violence advocates)? Probe: Who else could use the tool? Could it be used to build capacity (eg, training) or standardize information sharing?

### Feasibility Criteria

As recommended by Thabane et al [44], we established formal Stop or Go criteria to evaluate the success of the feasibility study. A Stop outcome states that the subsequent large-scale trial is not feasible, while a Go outcome means that the study can continue, with or without modifications [44]. The outcomes will be determined after assessing the study data. We will stop if (1) no positive change approaching significance in any of the primary outcomes in SC+: survivors’ housing knowledge, housing self-efficacy, or staff trust, relative to SC, is observed; or (2) survivors, on average, do not intend to continue the use of the tool, as measured by scores of 2=Disagree or 1=Strongly Disagree on the Likert scale. Criteria #1 and #2 will help us determine whether SC+ shows promise of a positive effect and acceptability (as determined by intent to use) to warrant a subsequent intervention trial to a larger group. If either Stop criterion is met, then the full-scale study is not feasible.

If both Stop criteria are not met, then we can move to a Go, that is, continue to a large-scale trial. Modifications will be needed if (1) 75% or more of the questions in the survey are left unanswered, (2) 75% or more of potential survivor participants do not join the study, or (3) less than 95% of enrolled survivors do not complete both the baseline and follow-up surveys. This retention rate is informed by the retention rates of the web-based myPlan evaluation, which hovered around 94% at 6 months and 93% at 12 months [27]. The Go criteria will help us determine whether the study could enroll and engage sizable participation, as illustrated by the rates of recruitment, retention, and response. 

### Statistical Analysis

Multiple linear regression models will be used to test the difference-in-difference in means of housing knowledge, housing self-efficacy, and staff trust. The inclusion of independent variables in the models will be determined by prior tests assessing whether any significant differences exist in outcome variables by demographic and moderating variables. The outcomes of interest will also be input as independent variables to determine the association between housing knowledge, housing self-efficacy, and staff trust.

Sample size, the rates of recruitment, retention, and response, and timeline will be recorded. Ease of implementation and outcome appropriateness will be collected through open-ended questions in individual interviews with the key informants, while acceptability and perceived usability will be collected through a mix of Likert scale and open-ended questions in the surveys and interviews.

To analyze the qualitative data, the written answers from the participant surveys and the transcripts from the key informant interviews will be inductively coded using a constant comparative method. Two coders will separately code the transcripts and then compare and iterate for agreement. Interrater reliability will be calculated as the number of codes in agreement, in proportion to the total number of codes [45]. We will iterate our codes until we achieve a minimum 80% agreement on 95% of our code set [45].

### Ethical Considerations

The study received approval from the Johns Hopkins Bloomberg School of Public Health Institutional Review Board (18116). To minimize the risk of a confidentiality breach of the participant’s personally identifiable information, we closely followed the World Health Organization [46] guidelines on domestic violence research, which includes the use of unique study identification numbers for participants. Consent will be collected in two ways. First, a participant clicking yes on the consent form to continue to the baseline survey will constitute consent. The study will not obtain participant signatures to protect confidentiality. Participants will receive US $10 for each completed survey, US $20 total, in e-gift cards. Second, the study will ask for oral consent from the key informants at the start of their interviews. Key informants will receive a US $20 e-gift card for participating in the individual interviews.

## Results

Recruitment for the SC arm began in March 2022 and completed in April 2023. Of the prospective participants, 75 participants were screened, 44 participants were deemed eligible, 35 participants were enrolled (ie, consented to the study), and 23 participants completed both baseline and follow-up surveys. Initially, we had sought to attain 50 completed surveys, 25 in each arm. However, the recruitment timeline for SC took longer than anticipated, and the enrollment numbers were lower than expected. Following 1 year of recruitment for SC that resulted in 23 completed surveys, we sought to expedite the recruitment timeline for SC+. Recruitment for the SC+ arm started in January 2024 and is anticipated to end by May 2024. After SC+ arm recruitment ends, we plan to recruit, conduct, and complete the key informant interviews in June 2024. We subsequently expect to complete the study and be ready to analyze the results by the end of June 2024.

## Discussion

### Hypothesized Findings

Among the results, we anticipate that SC+ will show a positive change relative to SC on at least one of the primary outcomes: survivors’ housing knowledge, housing self-efficacy, or staff trust. While the change may or may not be statistically significant, we are looking for the direction of change as an indication of possible future change in a large-scale trial. We also expect that most survivors in the SC+ group will deem the tool usable and acceptable. Overall, we anticipate achieving a Go outcome on feasibility for subsequent testing, likely with modifications to processes such as recruitment and retention.

### Comparisons to Prior Work

Only a handful of digital (mobile and nonmobile) solutions address the needs of survivors of domestic violence. In the United States, these solutions fall into three main categories. First is an educational and practical assistance category in which solutions educate users on domestic violence, engage users in safety planning, and connect users to resources, that is, myPlan, Aspire News, and bMORESafe [25-27]. Second is an emergency assistance category in which solutions alert survivors’ preprogrammed networks of the need for help, that is, bSafe, Kinetic Global (formerly Lifeline Response), and Circle of 6 [24-26]. Third and less common is a documentation category that empowers survivors with the capability of storing evidence of the abuse for later use. The National Network to End Domestic Violence had previously launched DocuSAFE for this purpose, but unfortunately, as of October 2023, the app is no longer functioning [47,48].

Regardless of category, studies of digital domestic violence solutions have demonstrated promising outcomes, particularly in addressing the safety planning needs of survivors [25-27]. The myPlan safety planning app, for instance, has been associated with reductions in intimate partner violence experience and risk of suicide [26]. Moreover, the app’s predecessor, the myPlan website, has been shown to increase survivors’ knowledge and perceived helpfulness of safety behaviors [27]. Survivors using the website were also more likely to have left their abusers than survivors who did not use the myPlan website over the 1-year study period [27]. All things said, none of these digital interventions address the housing needs of survivors. To the best of our knowledge, this will be the first time a digital housing tool for survivors of domestic violence will have been developed or studied in the United States.

### Limitations

Among the study’s limitations, first, the case managers will not be entirely blinded to the participants in the SC+ group. Case managers will be encouraged to use the tool with all survivors regardless of their enrollment status. However, if a survivor receives the tool from the study team and informs their case manager, then the case manager will know that the survivor has enrolled in the study, thereby potentially leading to differential treatment of survivors. Still, participants will not know of their arm assignments, thereby minimizing the risk of such knowledge influencing participant outcomes. Second, the specificity of the study’s setting in a community-based domestic violence center that helps survivors with disabilities, in New York City, may limit the applicability of results to other settings. Nevertheless, we believe that domestic violence and disability organizations in other urban areas could still gain from the findings and lessons of this study.

### Future Directions

#### Considerations

When implementing the SC arm, the study team encountered several delays with data collection that have been highlighted here for future consideration.

To start with, the study was conducted during the pandemic, at a time when nonprofits, like the partner site, were experiencing significant labor shortages and funding constraints [49,50]. At the same time, demand for services continued to increase, leading more nonprofits to report a decrease in their ability to provide services [51]. As such, the study’s data collection efforts often inadvertently competed with the staff’s full workload. In turn, the crisis context of domestic violence work also may have prevented the staff from fully participating in the study as they had hoped.

Further delaying data collection were unanticipated enrollment issues. Halfway through SC recruitment, we discovered about half of the SC participants were not truly eligible for the study. We infer that some participants had forwarded the study information to friends and family, who then falsely answered the screening questions to participate in the study. While we subsequently removed those faulty participants, we also extended the SC recruitment timeline to make up for the loss.

Finally, the study team had not accounted for the time it would take to obtain the appropriate site approvals, nor had we prepared for an unexpected change in case management systems.

#### Recommendations

Regardless of the issues we may have encountered, we continue to advocate for research in community settings. Community-placed research, such as this study, allows researchers unparalleled insight into the operations of community settings, which would only strengthen researchers’ efforts to effect sustainable change. Thus, to help researchers prepare for work in similar settings, we offer a few recommendations from our experience.

First, outline a partnership agreement with the community partner before any research begins. The agreement will delineate the expectations of time, resources, and approvals and will ensure that all parties have a shared understanding of and buy-in for the research study. We did not engage in a partnership agreement for this study.

Second, ensure that you have champions with some decision-making authority within the partner site to advocate for your partnership.

Third, even with the right champions as we had, maintain regular communication with the partner site to proactively preempt or address any issues as they arise. For instance, as in our case, the study team could have suggested actively comparing the participants’ emails sooner to prevent false participants.

Fourth, recognize how the crisis context of a community-based domestic violence organization may impact participation in the study. There is a growing body of evidence that calls for researchers to be aware of the multilevel forms of trauma existing within gender-based violence organizations and how the trauma may present itself [52]. This trauma may show up as last-minute meeting changes to address survivors’ crises or secondary trauma from direct service provision [52].

Finally, be ready to adapt. Following our observations with the SC arm, for instance, we subsequently adapted and shortened the recruitment timeline for the SC+ arm.

### Conclusions

While some of the issues, like the crisis context, will remain unavoidable, we can proactively take steps to ensure the successful implementation of future research within similar community settings. Future researchers could consider using community-based participatory research (CBPR) approaches, for instance, to guide their transition from conducting community-placed to community-based research. Central to CBPR is the equitable involvement of community stakeholders and researchers in all steps of the research [53,54]. Equitable involvement can look like shared decision-making, expertise, and ownership at each step by both the academic and community partners [53,54]. While other approaches exist, CBPR has gained much attention for its association with positive health outcomes for communities [54,55]. Still, little is known about the organizational capacity to support these outcomes [54,55], and we would remind researchers to be mindful of the capacity required to build a successful CBPR. Considerations aside, through this protocol, we aspire to encourage more research at the intersection of domestic violence, housing, and technology.

## Appendix

Multimedia Appendix 1 Presentation of the feasibility study.

## Abbreviations

CBPR: community-based participatory research
CHW: community health worker
DHS: Department of Homeless Services
DVHF: Domestic Violence Housing First Program
HF: Housing First
PSH: permanent supportive housing
RCT: randomized controlled trial
REDCap: Research Electronic Data Capture
SC: standard care
SC+: Standard care plus

## Data Availability

The datasets generated or analyzed during this study are not publicly available due to the sensitive nature of the study but are available from the corresponding author upon reasonable request.
