# Supplementary material for: Assessing a Digital Tool to Screen and Educate Survivors of Domestic Violence on Affordable Housing Programs in New York City: Protocol for a Mixed Methods Feasibility Study
Source: JMIR Res Protoc. 2025 May 2;14:e63162. doi: 10.2196/63162 (PMC12084769; doi:10.2196/63162)
Supplement: Multimedia Appendix 1 [file resprot_v14i1e63162_app1.pdf]

JOHNS HOPKINS
BLOOMBERG SCHOOL
of PUBLIC HEALTH

Assessing a Digital Tool to Screen and Educate Domestic Violence Survivors on Affordable Housing Programs in New York City: A Feasibility Study

DrPH candidate: Jennifer Tan
Advisor: Dr. Michelle Kaufman

May 18, 2022

Background: Macro-level

- There is a strong association between domestic violence (DV)/intimate partner violence (IPV), disability, housing instability (HI), and homelessness (1-6)
  - Women survivors of IPV are **4x more likely** to experience housing insecurity than women who have not experienced IPV (7)
  - **More than a third (38 percent)** of women become homeless immediately after leaving their abuser (8-9)
  - Lifetime IPV prevalence: **1 in 4 women and 1 in 10 men** (10)
  - Adults with disabilities, in the U.S. and worldwide, are at higher risk of IPV than adults without disabilities (12)
  - Having a disability further increases a survivor's vulnerability to housing insecurity and homelessness (11)

Background: Micro-level

- "It can be hard to explain to residents why or why they would not qualify for housing certificates"
- "Sometimes clients in DV shelter assume they will automatically be eligible for housing subsidies."
- "Whenever anything happens, the housing specialist gets blamed. I'd like something that validates what I say to clients."

Knowledge

Expectations

Trust

Source: Interviews with housing specialists

Current State & Gaps

- Common survey tool used in NYC

Household Composition

Household Composition:	Single
Is the Applicant pregnant?	
Number of children in household under 18:	0
Number of adults (18 and over) in household, including head of household, partners/spouses, and other adults:	1

Housing/Homeless Status

Homeless History as of 06/17/2020 from Administrative Systems

☐ 12 Months Continuous ☐ 4 Episodes in 3 Years

Data Source	System Match - ID#	Currently In Shelter	Past 1 Year	Past 2 Years	Past 4 Years
DHS - Singles & Family	NO				
HASA	NO				
Supportive Housing	YES - 181353				
TOTAL		NO	0	0	0

Note: Homeless episodes with overlapping days where a client is reported to be in different homeless systems at the same time will be counted once. The total days homeless may not reflect the cumulative days listed in the homelessness and housing history grid.

Is your client currently homeless?	Yes	User Entry
Has your client been homeless for at least one continuous year or experienced at least four episodes of homelessness in the past three years?	No	User Entry
Has your client been homeless for 365 days out of the last two years?	No	System
Has your client been homeless for 90 days out of the past year in a DYCD, DHS shelter or Street Homeless?	No	System
Has your client been homeless for two out of the last four years?	No	System
Is your client currently a DHS client (including street outreach)?	No	System
Is your client currently in a shelter?	No	System
Is your client currently staying in a DHS drop-in center or safe haven?	No	User Entry
Has your client experienced an eviction in NYC sometime in the year before entering DHS shelter OR currently in the process of being evicted OR were evicted in NYC within the last 12 months?	No	
When the individual or family thinks of ALL their family and friends, in NYC and outside the City, is there anyone they feel there is a strong connection with and would be a good place to stay?	No	

Strengths

- Systematic way of finding out what clients are eligible for and why

Gaps

- Clients do not have direct access to the information which can be disempowering
- Clients have anecdotally expressed confusion over the subsidies that they're eligible for and why, despite staff help
- Staff burden

Digital intervention (“tool”)

1. Web-based tool that screens users for NYC housing programs and options in 5-minutes or less, all from the ease of their phones. Aims to:
   - Facilitate **direct client access to information** on programs,
   - Explain **reasons for potential program eligibility**, and
   - Build **trust in staff**.

2. Initial audience:
   - Housing specialists and case managers
   - Survivors of domestic violence

3. Does not guarantee access to housing nor does it include all housing programs; instead, features the programs most frequently recommended by housing specialists in an accessible manner.

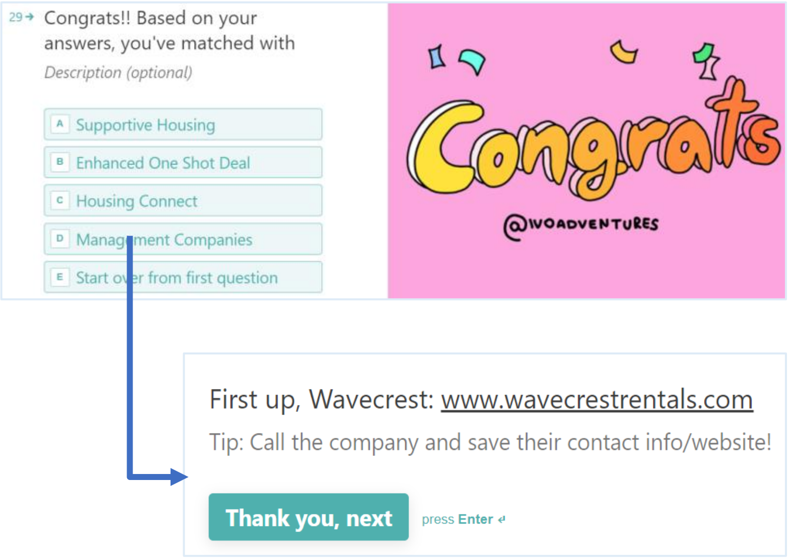

Study Aims

- Study Aims: To determine whether adding the tool to the site's services would be feasible, to explore users' perspectives on the tool, and to inform future iterations of the tool.

- Two treatment groups:
  - Use of the tool (Standard Care Plus SC+)
  - No use of the tool (Standard Care SC)

Research Questions

1. What is the initial treatment effect of SC+, as compared to SC, on survivors' housing knowledge, housing self-efficacy, and staff trust?

2. What is the estimated sample size needed for a full intervention trial? What is the estimated timeline needed to reach this sample size?

3. Are the expected rates of recruitment and retention achieved?

4. Are the study's processes and instruments feasible for a full intervention trial?

5. Is the tool acceptable and usable by survivors and staff?

Hypothesis

- **Hypothesis:** SC+ will show promise of a significant and greater positive change on survivors' housing knowledge, housing self-efficacy, and staff trust relative to SC.

- **Pathway to Change:** Increase in knowledge is expected to lead to an increase in self-efficacy, and vice versa. Either increase in knowledge or self-efficacy is expected to lead to an increase in staff trust, and vice versa.

- **Supporting Evidence:**
  - myPlan website RCT saw increases in knowledge of safety behaviors (13)
  - Help received in housing during shelter stay was associated with increases in survivors' self-efficacy and perceived shelter helpfulness (14)

Theoretical Framework

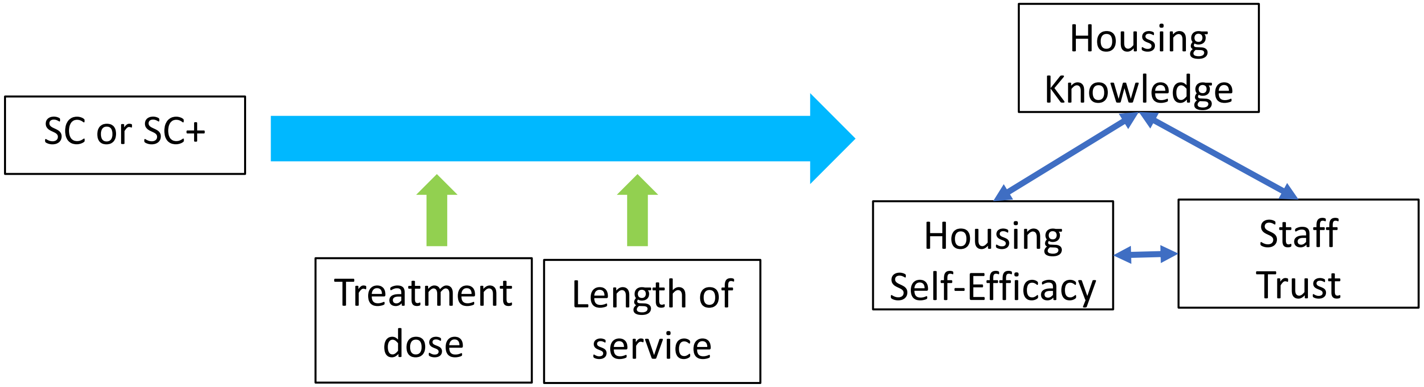

Study Procedures

- Study Setting: Community-based domestic violence center
- Participants: Survivors and staff
- Historical cohort design: Start by recruiting for SC, followed by recruiting for SC+
- Once enrolled, survivors will complete two surveys in two-four weeks:
  - t=0 (baseline): Baseline survey prior to treatment
  - t=2 weeks (follow up): Follow up survey two weeks later
- At the end of study, the case managers and Program Director will be invited to participate in (optional) individual 60-min interviews
- Incentives: $10 e-gift card for each completed survey ($20 total); $20 for case managers and Program Director, regardless of interview completion

Study Procedures (continued)

SC

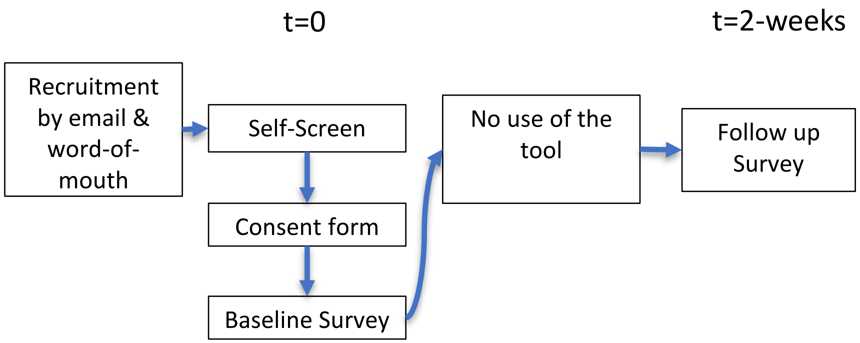

SC+

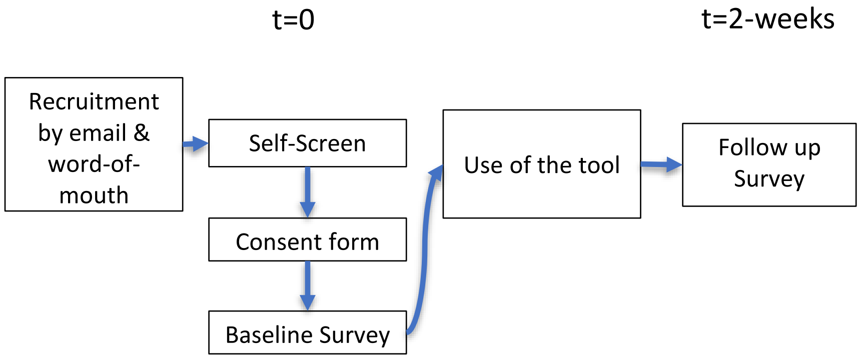

Screening Criteria for Client Participants

- Consent to participate in the study
- 18-years or older
- Seeking assistance from the partner site
- Have access to a device with web-browsing capability
- Looking for housing
- Has a safe email account
- Can read English without assistance, including translation or interpretation assistance

Protection of Human Subjects

Pre-Study

- CITI Human Subjects Research course for JHSPH study team only
- IRB approval
- Disclosure of conflict of interest

Consent

- Unique ID#s for participants
- Key file stored in encrypted drive and deleted at end of study
- Communication of study information, including benefits and risks
- Waiver of documentation of informed consent

Staff Interview

- Use of fake names for survivors
- Recording stored on encrypted drive and deleted after transcription check
- Communication of study information, including benefits and risks
- Oral consent

Contributions

- No previously published studies on digital solutions addressing the housing needs of survivors, to the best of our knowledge.
- A feasibility study would inform and ensure successful implementation of further research in this area.
- The current study significantly contributes to the field of domestic violence and its body of research by exploring the feasibility of a web-based intervention to address the housing needs of survivors.

JOHNS HOPKINS
BLOOMBERG SCHOOL
of PUBLIC HEALTH

Thank you!

References (in order of appearance)

1. Baker, C. K., Billhardt, K. A., Warren, J., Rollins, C., & Glass, N. E. (2010). Domestic violence, housing instability, and homelessness: A review of housing policies and program practices for meeting the needs of survivors. Aggression and Violent Behavior, 15(6), 430-439. https://doi.org/10.1016/j.avb.2010.07.005

2. Daoud, N., Matheson, F. I., Pedersen, C., Hamilton-Wright, S., Minh, A., Zhang, J., & O'campo, P. (2015). Pathways and trajectories linking housing instability and poor health among low-income women experiencing intimate partner violence (IPV): Toward a conceptual framework. Women & Health, 56(2)10.1080/03630242.2015.1086465

3. Jasinski, J. L., Wesely, J. K., Mustaine, E., & Wright, J. D. (2005). The experience of violence in the lives of homeless women: A research report. https://www.ojp.gov/pdffiles1/nij/grants/211976.pdf

4. Rollins, C., Glass, N. E., Perrin, N. A., Billhardt, K. A., Clough, A., Barnes, J., Hanson, G. C., & Bloom, T. L. (2012). Housing instability is as strong a predictor of poor health outcomes as level of danger in an abusive relationship. Journal of Interpersonal Violence, 27(4), 623-643. 10.1177/0886260511423241

5. Sullivan, C. M., López-Zerón, G., Bomsta, H., & Menard, A. (2018). 'There's just all these moving parts:' Helping domestic violence survivors obtain housing. Clinical Social Work Journal, 47(2), 198-206. 10.1007/s10615-018-0654-9

6. D'Ercole, A., & Struening, E. (1990). Victimization among homeless women: Implications for service delivery. Journal of Community Psychology, 18.

7. Pavao, J., Alvarez, J., Baumrind, N., Induni, M., & Kimerling, R. (2007). Intimate partner violence and housing instability. American Journal of Preventive Medicine, 32(2), 143-146. 10.1016/j.amepre.2006.10.008

8. Baker, C. K., Cook, S. L., & Norris, F. H. (2003). Domestic violence and housing problems: A contextual analysis of women's help-seeking, received informal support, and formal system response. Violence Against Women, 9(7), 754-783. 10.1177/1077801203253402

References (in order of appearance)

9. Gezinski, L. B., & Gonzalez-Pons, K. M. (2019). Unlocking the door to safety and stability: Housing barriers for survivors of intimate partner violence. Journal of Interpersonal Violence, 886260519851792. 10.1177/0886260519851792

10. D'Inverno, A. S., Smith, S. G., Zhang, X., & Chen, J. (2019). The impact of intimate partner violence: A 2015 NISVS research-in-brief. Atlanta, GA: National Center for Injury Prevention and Control, Centers for Disease Control and Prevention.

11. Adams, E. N., Clark, H. M., Galano, M. M., Stein, S. F., Grogan-Kaylor, A., & Graham-Bermann, S. (2021). Predictors of housing instability in women who have experienced intimate partner violence. Journal of Interpersonal Violence, 36(7-8), 3459-3481. 10.1177/0886260518777001

12. Bonomi, A. E., Anderson, M. L., Cannon, E. A., Slesnick, N., & Rodriguez, M. A. (2009). Intimate partner violence in Latina and non-Latina women. American Journal of Preventive Medicine, 36(1), 43-48. 10.1016/j.amepre.2008.09.027

13. Glass, N. E., Perrin, N. A., Hanson, G. C., Bloom, T. L., Messing, J. T., Clough, A. S., Campbell, J. C., Gielen, A. C., Case, J., & Eden, K. B. (2017). The longitudinal impact of an internet safety decision aid for abused women. American Journal of Preventive Medicine, 52(5), 606-615. 10.1016/j.amepre.2016.12.014

14. Sullivan, C., & Virden, T. (2017). An eight state study on the relationships among domestic violence shelter services and residents' self-efficacy and hopefulness. Journal of Family Violence, 32(8), 741-750. 10.1007/s10896-017-9930-7
